# Job vacancy at the European Centre for Disease Prevention and Control (ECDC)

**DOI:** 10.2807/1560-7917.ES.2016.21.41.30371

**Published:** 2016-10-13

**Authors:** Eurosurveillance editorial team

**Affiliations:** 1 European Centre for Disease Prevention and Control (ECDC), Stockholm, Sweden

**Keywords:** public health

The European Centre for Disease Prevention and Control (ECDC) invites applications for the following position:

• Senior Expert Public Health Emergency Preparedness Unit

For detailed information on the job description, requirements and the application procedure, please visit the ECDC website at: http://ecdc.europa.eu/en/aboutus/jobs/Pages/JobOpportunities.aspx

